# Supplementary material for: Human mobility at Tell Atchana (Alalakh), Hatay, Turkey during the 2nd millennium BC: Integration of isotopic and genomic evidence
Source: PLoS One. 2021 Jun 30;16(6):e0241883. doi: 10.1371/journal.pone.0241883 (PMC8244877; doi:10.1371/journal.pone.0241883)
Supplement: S1 File — (DOCX) [file pone.0241883.s003.docx]

# **S1 File. The chronology of the burials: detailed analysis of stratigraphy and radiocarbon dating**

It has become standard practice in aDNA studies to radiocarbon date many of the individuals with good endogenous DNA preservation. This step is crucial, as a reliable interpretation of population genomic analyses can only be ascertained as long as no samples dated to the wrong time period interfere with the datasets. This means that currently as a mere byproduct of aDNA analyses, large radiocarbon datasets of burials are generated, a find category that is usually not a prime target for direct dating in archaeology – a neglect that is justified, if one considers that a radiocarbon date derived from a well-stratified unit within a settlement can contribute more to the understanding of a site as a whole than a date derived from a single grave. Radiocarbon dates of many graves from one and the same site, on the other hand, hold the power to substantially improve the understanding of the whole burial corpus, especially if the graves are not accompanied by grave goods of chronological significance and/or lack clear stratigraphic associations, as is the case with the extra-city cemetery at Alalakh [1-3].

While the burials in the extra-city cemetery located in Squares 45.71 and 45.72 can be placed within the wider stratigraphic sequence of the settlement – with a first phase of burial activity after the destruction of the MBA casemate city wall but before the erection of a kitchen/workshop complex, and a second phase of burial activity, to which only five graves are dated (L04-2050, L03-3017 = the Plastered Tomb, L03-3011, L03-3019, and L03-3013/3016 = ALA095), after the destruction of the kitchen/workshop complex during LB I – those within Squares 45.44 and 45.45 lack any associations with architecture [1, 4]. Most burials from Squares 45.44 and 45.45 (65 out of 80) have been tentatively paralleled with the second, LBA phase of burial activity in Squares 45.71 and 45.72 [1]. However, it needs to be stressed here that the stratigraphically clear separation between the first and second phases of extra-city burials in Squares 45.71 and 45.72 does not exist in Squares 45.44 and 45.45, with the burials in the latter squares occurring continuously throughout the excavated sequence.

A comparison between the archaeological dating (stratigraphy and grave goods) and the AMS ^14^C-dates of the 33 individuals from Alalakh (6 first published here and 27 published in Skourtanioti et al.) [5] reveals two significant discrepancies, if we assume the common rough relative period-absolute date correlation for the northern Levantine area that is also generally accepted for Alalakh of MB I c. 2000-1800 BC, MB II c. 1800-1650/1600 BC, LB I c. 1650/1600-1400/1350 BC, and LB II c. 1400/1350-1200 BC [6, 7] (Fig 3).

On a superficial level, the radiocarbon dates of the individuals conform with the archaeological dating of the graves that places them in the MBA-LBA, so roughly between 2000-1200 cal BC, but if we zoom in, it becomes apparent that 1) all individuals from the extra-city cemetery that were ^14^C-dated (with the exception of the Plastered Tomb) date before 1600 cal BC, hence the MBA in relative terms, while archaeologically, the main period of use of the cemetery is supposedly in the LB I; and, that 2) some of the burials in the extra-city cemetery likely date back well into the MB I.

(1) We believe the discrepancy between archaeological dating and radiocarbon dating stems from the lack of clear stratigraphical connections between Squares 45.44/45.45 and Squares 45.72/45.71. The burials in Squares 45.44 and 45.45 seem all to belong to the older/first phase of the extra-city cemetery, so to Period 7 (MB II) or before. In the time of renewed burial activity in the LB I that is definitely present in Square 45.71 – confirmed by both stratigraphy and radiocarbon dating of the Plastered Tomb – there were either no new burials in Squares 45.44 and 45.45 or (less likely), they are all eroded and therefore destroyed. It seems unlikely that by chance only those individuals from Squares 45.44 and 45.45 belonging to the MBA were ^14^C-dated, as a total of 15 individuals from these two squares of the extra-city cemetery were radiocarbon dated from all of the tentative phases of the cemetery established via burial depths (Periods 7-4) [1]. The tentative phases (Local Phases 6-1) of the extra-city cemetery in Squares 45.44 and 45.45 established by Ingman [1] may well be correct – radiocarbon dates simply span too many years to allow any claims about such fine-grained phasing – but they may need to be pushed backwards in time to before 1600 cal BC and correlated with Periods 7 and earlier. If this is the case, burial activity in the extra-city cemetery would have stopped around the time the Level VII palace was destroyed. At some point after this, burial activity resumed on the eastern slope, but only on a limited scale in Square 45.71 and perhaps also Square 45.72. This limited activity is represented by the aforementioned five graves dated archaeologically to Local Phase 1, including the Plastered Tomb, in Period 4 in the late LB I [4]. The existing three radiocarbon dates from three of the four individuals in the Plastered Tomb support its archaeological dating. What is not possible to answer from the available radiocarbon dates is when burial activity in Squares 45.71 and 45.72 resumed during LB I after the destruction of the Local Phase 2 kitchen/workshop context, as the Plastered Tomb is archaeologically dated to the very end of burial activities on the eastern slope of the tell, and the only other burial stratigraphically belonging to the LB I phase of the cemetery in Square 45.72 that was radiocarbon dated clearly falls into the MBA (ALA095: Locus L03-3016, cal BC 1913-1756, 2σ). This tooth belongs to a heap of bones of minimally three individuals in a secondary position on top of a single primary burial. There are two possibilities to explain the early date for ALA095: either Square 45.72 was, like Squares 45.44 and 45.45, only used for burials during the first phase of the cemetery in the MBA or the primary burial in Locus L03-3016 actually dates to the LB I period of the cemetery, but the comingled remains on top, and with them the tooth that was radiocarbon dated, belong to the earlier phase of the cemetery. While digging the grave pit for the primary burial in the later phase, some older burials in the same location could have been disturbed. The bones from these older burials could have been collected and simply reburied in a heap on top of the later primary burial, a phenomenon that is documented throughout the extra-city cemetery [1, 3].

(2) Until now it was thought that the earliest burials of the extra-city cemetery dated to the MB II. It seems this idea needs to be revised substantially. At least five of the radiocarbon dates indicate a date in the MB I or at the latest in the first three decades of the MB II (ALA024, ALA015, ALA084, ALA009_1, and ALA035). If the dates are correct, this means that burial activity on the eastern slope already started before Period 8 at Alalakh. This would make the extra-city cemetery one of the oldest features that has been excavated at Tell Atchana to date. We generally consider the radiocarbon dates of the samples to be correct and not impacted by marine or freshwater reservoir effects that could account for older dates [8, 9]. Alalakh lies inland within the Amuq Valley, and therefore large quantities of marine diet intake are unlikely. However, the stratigraphy of Area 3 and the final phasing and dating of these contexts, including a full integration with other radiocarbon dates from these squares, is currently in progress, so these remain suggestions for the time that will be explored further as analysis progresses.

In contrast to the results of radiocarbon dating from the extra-city cemetery, the intra-city burials show a higher level of concordance with the archaeological dating, which is hardly surprising, considering the burials’ positioning within the stratigraphy of the settlement. Only three (ALA016, ALA020, and ALA131) out of 13 intra-city individuals show unexpected results: the 2σ date range for individual ALA020 of cal BC 1502-1395 suggests an LB I (Period 4) date for this individual, rather than the preliminary LB II (Period 2-1) date indicated by the burial context. ALA020 (Square 44.86, Local Phase 2, Loci 18 and 20) is a single primary burial in a simple pit grave that was dug into a debris layer; the contexts in this square are still under study, so the initial LB II date is tentative. Individual ALA016 represents a similar situation, with a ^14^C date that suggests an older date – cal BC 1617-1506, 2σ (early LB I) – than the stratigraphic positioning of the burial in Square 32.54 within the destruction deposits of Building 2006-4 (Local Phase 2d), dated to late LB I (Period 4), and under the intact floor of the next building phase (Local Phase 2c), dated to the early LB II (Period 3) [10]. The stratigraphic positioning of both burials, especially ALA016 is clear, and we cannot resolve the discrepancy in radiocarbon and archaeological dating.

Individual ALA131 is a single primary burial from the intra-city cemetery in Area 4 that has been under excavation since 2015, and so far 28 burials have been fully or partially uncovered. The burials were concentrated in a small area of abandoned buildings and were stratigraphically dated to Periods 6-4, which means the LB I period, with the possibility that a few burials might date to the MB II/LB I transition [3]. Of the five burials from this Area that were radiocarbon dated, four fall roughly into this anticipated timeframe (ALA130, ALA135, ALA136, and ALA138), while the LB II date for ALA131 (1428-1287 cal BC 2σ) is unexpectedly late and clearly set apart from the sequence of the other four burials by at least 120 years. The date raises the question of whether burial ALA131 should be seen as a proper part of the intra-city cemetery or if this individual was buried in this area only later, after burial activity had already stopped here for some centuries. The only option to gain further clarity would be to ^14^C-date more of the burials in the Area 4 cemetery.

# **References**

1. Ingman T. The Extramural Cemetery at Tell Atchana, Ancient Alalakh and GIS Modeling. In: Maner Ç, Horowitz M, Gilbert A, editors. Overturning Certainties in Near Eastern Archaeology: A Festschrift in Honor of K Aslıhan Yener. Leiden: Brill; 2017. p. 245-58.

2. Ingman T. Mortuary Practices and GIS Modeling at Tell Atchana, Ancient Alalakh. In: Yener KA, Ingman T, editors. Alalakh and its Neighbors: Proceedings of the 15th Anniversary Symposium at the New Hatay Archaeology Museum, June 10-12, 2015. Leiden: Peeters; 2020. p. 389-406.

3. Ingman T. Identity and Changing Funerary Rituals at Tell Atchana, Alalakh: Mortuary and Isotopic Analyses. Istanbul: Koç University; 2020.

4. Yener KA, Yazıcıoğlu GB. Excavation Results. In: Yener KA, editor. The Amuq Valley Regional Projects: Excavations in the Plain of Antioch: Tell Atchana, Ancient Alalakh, Vol 1: The 2003-2004 Excavation Seasons. Istanbul: Koç University; 2010. p. 11-49.

5. Skourtanioti E, Erdal YS, Frangipane M, Balossi Restelli F, Yener KA, Pinnock F, et al. Genomic History of Neolithic to Bronze Age Anatolia, Northern Levant and South Caucasus. Cell. 2020;181:1158-75.

6. Morandi Bomacossi D. The Northern Levant (Syria) during the Middle Bronze Age. In: Killebrew AE, Steiner ML, editors. The Oxford Handbook of the Archaeology of the Levant: C 8000 – 332 BCE. Oxford: Oxford University Press; 2014. p. 414-33.

7. Sherratt S. Introduction to the Levant During the Late Bronze Age. In: Killebrew AE, Steiner ML, editors. The Oxford Handbook of the Archaeology of the Levant: C 8000 – 332 BCE. Oxford: Oxford University Press; 2014. p. 497-508.

8. Reimer PJ, McCormac FG. Marine Radiocarbon Reservoir Corrections for the Mediterranean and Aegean Seas. Radiocarbon. 2002;44(1):159-66.

9. Philippsen B. The Freshwater Reservoir Effect in Radiocarbon Dating. Heritage Science. 2013;1(1):24.

10. Akar M. Excavation Results. In: Yener KA, Akar M, Horowitz MT, editors. Tell Atchana, Alalakh, Vol 2: The Late Bronze II City, the 2006-2010 Excavations Seasons. Istanbul: Koç University Press; 2019. p. 11-75.
