# Supplementary material for: Human mobility at Tell Atchana (Alalakh), Hatay, Turkey during the 2nd millennium BC: Integration of isotopic and genomic evidence
Source: PLoS One. 2021 Jun 30;16(6):e0241883. doi: 10.1371/journal.pone.0241883 (PMC8244877; doi:10.1371/journal.pone.0241883)
Supplement: S2 File — (DOCX) [file pone.0241883.s004.docx]

# **S2 File. Discussion of the modern snail shells and their underlying geology**

We chose modern snail shells as sampling material to obtain local strontium baselines within the Amuq area, both for their abundance and their secure provenance. Sampling snails for both Alalakh and the Amuq Valley furthermore ensures a consistency that might improve the comparability of results. Unfortunately, there do not exist any other strontium studies of archaeological materials from other sites within the Amuq Valley with which we could potentially compare the ^87^Sr/ ^86^Sr values of the modern snail shells, although these will likely be part of future research, in which the use of ancient material stemming from drill cores is planned.

Altogether, we measured the ^87^Sr/ ^86^Sr ratios of nine modern snail shells from eight distinct locations within the Amuq (Fig 5, Table 6). The snail samples from Uydukent, Reyhanlı, and Kırıkhan were collected from the Holocene lake deposits that cover most of the Amuq Valley and on which Alalakh itself is also located. These three snail shells show ^87^Sr/ ^86^Sr ratios in the range of the samples from Alalakh or lower. The snail from Uydukent (0.70830), located closest to Alalakh, and, like Alalakh, in the center of the valley, is consistent with the ^87^Sr/ ^86^Sr ratios from Alalakh itself, while the Reyhanlı (0.70798) and Kırıkhan (0.70792) snails, located on the fringes of the valley basin close to other geological units on the valley slopes, have lower ^87^Sr/ ^86^Sr ratios.

The ^87^Sr/ ^86^Sr ratios of four samples from three locations on the southern margins of the Amuq Valley – Kamberli, Avcılar, and Hacıpaşa (2 samples) – scatter in the upper range of the Alalakh samples or above, from 0.70836-0.70881. Relatively higher strontium values are documented in the samples from Upper Miocene geological formations (Kamberli and Avcılar) and lower values in the samples from Pliocene Marine deposits (2 samples from Hacıpaşa). The two remaining samples were collected on the northwestern fringes of the Amuq Valley (Ceylanlı) and its northern outlet near the banks of the Kara Su River (Haydarlar). Geologically, Ceylanlı falls right on the border of four distinct units: biogenic and quaternary basalt deposits to the north, Paleogenic formations to the east, serpentine and peridotite to the south, and Paleozoic units to the west. Interestingly, the snail shell from this location shows a ^87^Sr/ ^86^Sr ratio of 0.70821, well within the values of Alalakh. The snail from Haydarlar, on the other hand, has the lowest documented ^87^Sr/ ^86^Sr ratio in our data series (0.70738). It is, like the snails within the valley basin, located on recent Holocene deposits, although these derive from alluvial deposits of the Kara Su River and not of the lake. The distinctly low isotopic signature of this snail seems to be influenced mainly by the basalt shields of Jurassic and Cretaceous age that are located along the slopes of the river valley, as basalt is reported to have very low initial Rb/Sr ratios of less than 0.704 [1].

# **References**

1. Price TD, Burton JH, Bentley RA. The Characterization of Biologically Available Strontium Isotope Ratios for the Study of Prehistoric Migration. Archaeometry. 2002;44(1):117-35.
