# Supplementary material for: Human mobility at Tell Atchana (Alalakh), Hatay, Turkey during the 2nd millennium BC: Integration of isotopic and genomic evidence
Source: PLoS One. 2021 Jun 30;16(6):e0241883. doi: 10.1371/journal.pone.0241883 (PMC8244877; doi:10.1371/journal.pone.0241883)
Supplement: S3 File — (DOCX) [file pone.0241883.s005.docx]

# **S3 File. Comparison between modern environmental ^87^Sr/ ^86^Sr ratios in Meiggs [1] and the samples in this study**

The ^87^Sr/ ^86^Sr range of 0.70780-0.70889 that we calculated for the Amuq catchment area using eight out of nine modern snail shells from the Amuq (excluding the outlier from Haydarlar) and the mean of the ancient snails and rodent teeth from Alalakh is more restricted than the range one obtains from the modern samples in Meiggs’s study (0.70727-0.70968, excluding the pine-needle outlier) [1]. A comparison between the ^87^Sr/ ^86^Sr ratios measured in the modern snail shells from this study and the modern samples from Meiggs’ [1] study, consisting of snail shells and plants, leads us to the conclusion that the plant samples are the main driver of this discrepancy.

Three snail shells from Meiggs’ study (samples G2.6, G2.1, and G1.8) come from Holocene lake deposits, and their ^87^Sr/ ^86^Sr ratios are compatible with the ones we obtained from the same geological unit (samples from Uydukent, Reyhanı, and Kırıkhan). Also, Meiggs’ sample G1.7 and our sample from Kamberli, both from Upper Miocene formations and generally located very close to each other, show consistent ^87^Sr/ ^86^Sr ratios. The same holds true for the modern snail shell from Alalakh in Meiggs’ study (AK01) and the ancient snail shells analyzed in our study. Therefore, while the snail shells generally show a good match with our samples, the plant ^87^Sr/ ^86^Sr ratios raise questions concerning the comparability of the measurements in Meiggs’ study in general. In particular, samples G6.9, G4.4, and G6.5, all from the northern fringes of the Amuq Valley, showed far higher ^87^Sr/ ^86^Sr ratios than we would expect. We cannot explain the discrepancy between Meiggs’ sample G6.9 and the snail shell from Haydarlar in our study. Both originate from Holocene alluvial deposits close to the Kara Su River and are located between the aforementioned basalt shields. While this setting explains the low ^87^Sr/ ^86^Sr ratio of the Haydarlar snail shell, the high ^87^Sr/ ^86^Sr ratio of the plant is divergent. This could either be explained by the influence of fertilizer (sample Gü-Sr [1]) or a more general disconnect caused by differing laboratory processing and analysis protocols between the two studies. As animal teeth and snail samples from Meiggs’ study are generally compatible with the results from our study, we conclude that likely only the plant samples are affected by fertilizer and/or issues arising during laboratory processing, and therefore decided to merge the snail shells and animal teeth from Meiggs’ study with our dataset to calculate local ranges for Alalakh and the Amuq Valley.

# **References**

1. Meiggs DC. Herding Practices, Urban Provisioning, and Human Mobility at Tell Atchana (Alalakh): 2009 Strontium Isotope (^87^Sr/^86^Sr) Results. Arkeometri Sonuçları Toplantası. Ankara: T.C. Kültür ve Turizm Bakanlığı, Kültür Varlıkları ve Müzeler Genel Müdürlüğü; 2010. p. 51-68.
